# Distinct Effects of Beta-Amyloid, Its Isomerized and Phosphorylated Forms on the Redox Status and Mitochondrial Functioning of the Blood–Brain Barrier Endothelium

**DOI:** 10.3390/ijms24010183

**Published:** 2022-12-22

**Authors:** Aleksandra V. Petrovskaya, Artem M. Tverskoi, Evgeny P. Barykin, Kseniya B. Varshavskaya, Alexandra A. Dalina, Vladimir A. Mitkevich, Alexander A. Makarov, Irina Yu. Petrushanko

**Affiliations:** Engelhardt Institute of Molecular Biology, Russian Academy of Sciences, 119991 Moscow, Russia

**Keywords:** blood–brain barrier, beta-amyloid modifications, redox parameters, mitochondrial function, bEnd.3

## Abstract

The Alzheimer’s disease (AD)-associated breakdown of the blood–brain barrier (BBB) promotes the accumulation of beta-amyloid peptide (Aβ) in the brain as the BBB cells provide Aβ transport from the brain parenchyma to the blood, and vice versa. The breakdown of the BBB during AD may be caused by the emergence of blood-borne Aβ pathogenic forms, such as structurally and chemically modified Aβ species; their effect on the BBB cells has not yet been studied. Here, we report that the effects of Aβ_42_, Aβ_42_, containing isomerized Asp7 residue (iso-Aβ_42_) or phosphorylated Ser8 residue (p-Aβ_42_) on the mitochondrial potential and respiration are closely related to the redox status changes in the mouse brain endothelial cells bEnd.3. Aβ_42_ and iso-Aβ_42_ cause a significant increase in nitric oxide, reactive oxygen species, glutathione, cytosolic calcium and the mitochondrial potential after 4 h of incubation. P-Aβ_42_ either does not affect or its effect develops after 24 h of incubation. Aβ_42_ and iso-Aβ_42_ activate mitochondrial respiration compared to p-Aβ_42_. The isomerized form promotes a greater cytotoxicity and mitochondrial dysfunction, causing maximum oxidative stress. Thus, Aβ_42_, p-Aβ_42_ and iso-Aβ_42_ isoforms differently affect the BBBs’ cell redox parameters, significantly modulating the functioning of the mitochondria. The changes in the level of modified Aβ forms can contribute to the BBBs’ breakdown during AD.

## 1. Introduction

Alzheimer’s disease (AD) is the most common neurodegenerative disease in the world [1]. The accumulation of beta-amyloid peptide (Aβ) in the brain and the formation of its toxic aggregates is a key factor for the initiation of the AD pathogenic cascade, triggering the hyperphosphorylation of the tau protein, neuroinflammation and neuronal death [2,3]. The progression of AD is associated with vascular abnormalities in the brain [4], in particular, with the breakdown of the blood–brain barrier (BBB) [5]. The BBB controls the exchange of substances between the peripheral blood flow and the brain, and it is composed of several cell types. The main barrier function is performed by brain endothelial cells [6]. The BBB provides Aβ clearance from the brain using the LRP1 receptor [7,8] and Aβ transport from the plasma to the brain using the receptor for advanced glycation end products (RAGE) [9,10]. There is a balance between these flows under normal conditions, whereas the BBBs’ breakdown impedes cerebral Aβ clearance [5,11,12] and contributes to the progression of AD. The cause of the breakdown of the BBB in AD may be the toxic effect of Aβ on the barrier’s components. In the in vitro mouse BBB model, Aβ leads to a BBB disruption and down-regulates the level of tight junction proteins (zonula occludens-1 (ZO-1), claudin-5 and occludin) in endothelial cells [13,14]. Neutralizing anti-RAGE antibodies prevent these changes [14]. The available data [14,15] suggest that the mechanism of an Aβ-induced BBB breakdown involves alterations in intracellular calcium and changes in the mitochondrial functioning. In contrast to neurons, endothelial cells transport Aβ by transcytosis to the other side of the cell membrane and do not accumulate Aβ inside. Thus, the effect of Aβ on the cells of the BBB may be explained by peptide binding to receptors. As we showed earlier [16], Aβ binding to receptors leads to a rapid change in the cell redox status. We believe that alterations in the Aβ-induced redox status may result in abnormal mitochondrial functioning in the BBBs cells.

Aβ was also shown to originate outside the brain, primarily in platelets [17]. In the experiments on mouse models, blood-borne Aβ enters the brain and triggers an Aβ-induced pathology, including the hyperphosphorylation of tau, neuroinflammation, and the formation of amyloid plaques [18,19]. At the same time, a prevailing form of blood-borne Aβ, accumulating in the mouse brain, is beta-amyloid 1–42 (Aβ_42_). Aβ_42_ is the main component of the senile plaques in AD. However, the intravenous injection of synthetic Aβ_42_ in the mouse did not initiate the pathology of AD [20,21]. Thus, an Aβ_42_ increase in the blood is insufficient for the progression of AD. Therefore, the presence of pathogenic forms of Aβ triggering the disease seems mandatory [22,23]. Aβ post-translational modifications can belong to the pathogenic forms of Aβ because these modifications significantly alter the effect of Aβ on the neuronal cells [23,24,25,26,27,28] and are an important factor in AD. The level of Aβ, containing isomerized Asp7 residue (iso-Aβ_42_) or phosphorylated Ser8 residue (p-Aβ_42_), varies with age, and these isoforms are of great interest [29,30,31]. Iso-Aβ_42_ accounts for more than 50% of all Aβ peptides in the brain of AD patients [31]. It has an increased neurotoxicity [25,27,32], and its intravenous administration accelerates cerebral amyloidogenesis in the brain of APP/PS1 AD model mice [20]. On the contrary, p-Aβ_42_ reduces the number of amyloid plaques in the hippocampus after an intravenous administration and demonstrates a reduced ability to form metal-dependent oligomers [26]. Thus, a disruption to the BBB in AD patients may be caused by the emergence of blood-borne Aβ pathogenic forms, but their effect on the BBBs cells has not been studied before.

We characterized the effects of Aβ_42_, p-Aβ_42_ and iso-Aβ_42_ peptides induced by a beta-amyloid treatment in the monomeric form on the mouse brain endothelial cells bEnd.3 to assess how Aβ isoforms modulate the key parameters of the BBBs cells, such as the redox status (reactive oxygen species (ROS), nitric oxide (NO) and reduced glutathione (GSH)), intracellular calcium and mitochondrial functioning. We found that p-Aβ_42_ affected these parameters modestly, whereas iso-Aβ_42_ caused maximum oxidative stress and induced the greatest cytotoxicity to the BBB endothelial cells.

## 2. Results

### 2.1. Iso-Aβ_42_ Induces the Highest Toxicity to the Blood–Brain Barrier Cells

**Figure 1:**
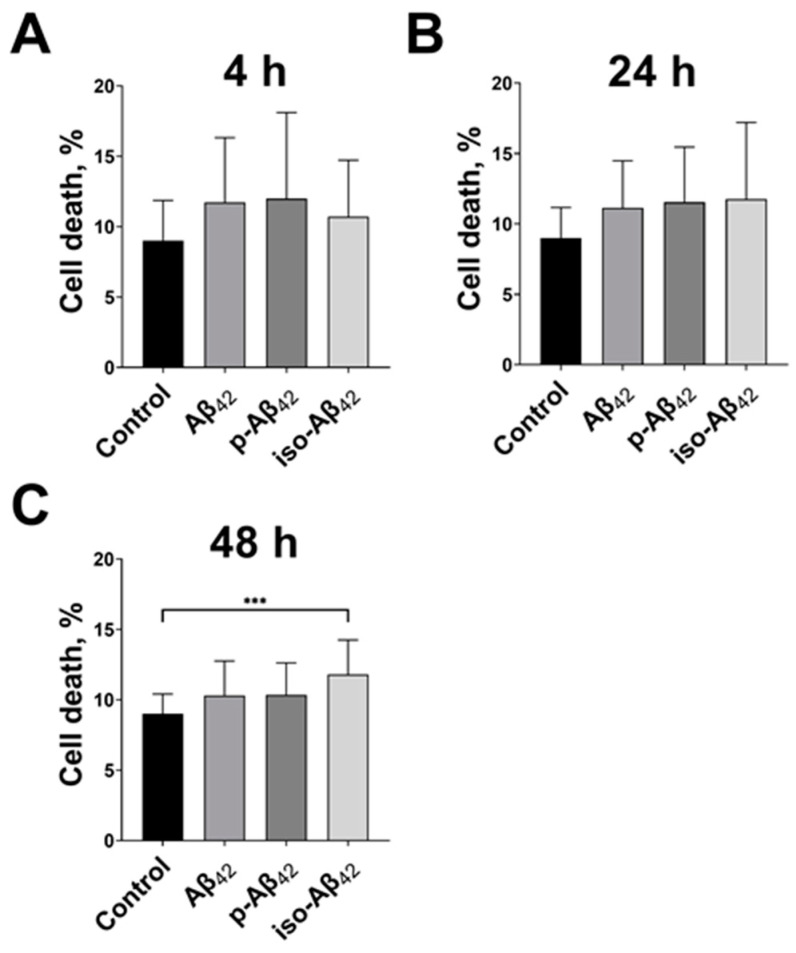
The effects of beta-amyloid isoforms on bEnd.3 cell death. The cells were incubated for (**A**) 4 h, (**B**) 24 h and (**C**) 48 h with 10 µM of Aβ_42_, p-Aβ_42_ and iso-Aβ_42_. The percentage of propidium iodide positive (dead) cells was analyzed by flow cytometry. The mean ± SD in 7 independent experiments in triplicates is shown in the figure. The statistical difference between experimental groups was analyzed by one-way analysis of variance using the post hoc Tukey test for multiple comparisons. ***—*p* < 0.001.

It is known that Aβ_42_-induced changes in the Ca^2+^ level are observed in the BBBs cells after several hours of incubation [14], although they are less sensitive to the Aβ_42_ toxic effect than neuronal cells [15,26]. To assess the effect of Aβ isoforms on the brain endothelium, bEnd.3 cells were incubated with Aβ peptides for 4, 24 and 48 h. A total of 10 μM of Aβ_42_ and p-Aβ_42_ did not affect the cell viability after 48 h of incubation (Figure 1). Under the same conditions, an iso-Aβ_42_ treatment led to an increase in the percentage of dead cells in the population by 25%, relative to the control (Figure 1).

### 2.2. Aβ_42_ and iso-Aβ_42_ Significantly Affect the Redox Status of the Blood–Brain Barrier Cells

**Figure 2:**
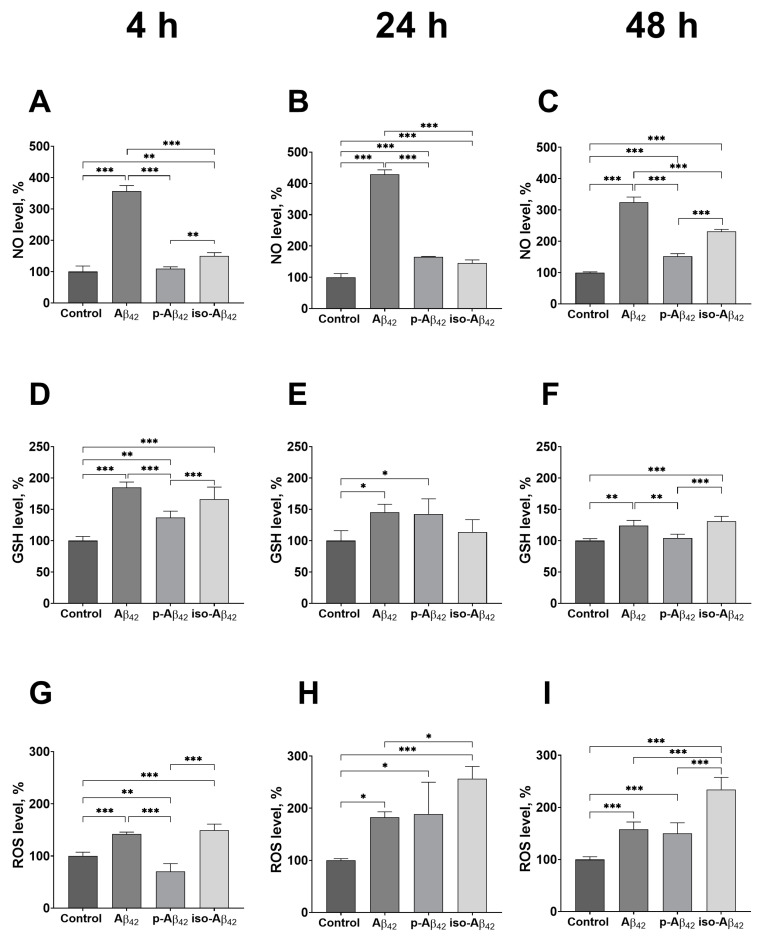
The effects of beta-amyloid isoforms on the redox parameters in bEnd.3 cells. Cells were incubated for 4 h (**A**,**D**,**G**), 24 h (**B**,**E**,**H**) and 48 h (**C**,**F**,**I**) with 10 µM of Aβ_42_, p-Aβ_42_ and iso-Aβ_42_. The level of intracellular nitric oxide (NO) (**A**–**C**), reduced glutathione (GSH) (**D**–**F**) and intracellular reactive oxygen species (ROS) (**G**–**I**) were analyzed by flow cytometry. All parameters were normalized to control. The values in the control samples were taken as 100%. The mean ± SD in 3 independent experiments in triplicates is shown in the figure. The statistical difference between experimental groups was analyzed by one-way analysis of variance using the post hoc Tukey test for multiple comparisons. *—*p* < 0.05, **—*p* < 0.01 and ***—*p* < 0.001.

To characterize the changes in the redox status in bEnd.3 cells treated with Aβ_42_ isoforms, the ROS, GSH and NO levels were assessed after 4, 24 and 48 h of incubation (Figure 2). After 4, 24 and 48 h of incubation with Aβ_42_, a 3.6-, 4.2- and 3.3-fold NO increase, respectively, was detected relative to the control (Figure 2A–C). In the presence of iso-Aβ_42_, this increase was less pronounced, being 1.5-, 1.5- and 2.5-fold, respectively (Figure 2A–C). The effect of p-Aβ_42_, in comparison with Aβ_42_ and iso-Aβ_42_, was less pronounced (Figure 2A–C): p-Aβ_42_ did not cause a NO increase after 4 h of incubation, and a comparatively low NO elevation (~1.6-fold) was observed after a 24 and 48 h exposure.

All three Aβ_42_-isoforms caused a GSH increase in the cells after 4 h of incubation (1.7-, 1.8- and 1.4-fold for Aβ_42_, iso-Aβ_42_ and p-Aβ_42_, respectively) (Figure 2D). After 24 h of incubation, the GSH level remained above the control for Aβ_42_ and p-Aβ_42_ (Figure 2E). At the 48 h time point, the Aβ isoforms-induced increase in the GSH level becomes less pronounced compared to 4 h of incubation. The GSH level in Aβ_42_- and in iso-Aβ_42_-treated samples exceeded the control values by 23% and 30%, respectively, and the GSH level in the p-Aβ_42_-treated samples did not differ from the control values (Figure 2F).

Incubation with Aβ_42_ and iso-Aβ_42_ led to an almost 1.5-fold increase in the ROS level after 4 h, while p-Aβ_42_ caused a decrease in the ROS level by 30% relative to the control (Figure 2G). After 24 h of incubation, an increase in the ROS level was observed for all three isoforms: in the presence of Aβ_42_ and p-Aβ_42_, it increased by a factor of 1.8, and in the presence of iso-Aβ_42_, it increased by a factor of 2.5 (Figure 2H). After 48 h, the mode of the ROS increase did not change (Figure 2I).

We analyzed the effects of Aβ isoforms on the endothelial nitric oxide synthase (eNOS) monomer/dimer ratio (Figures S1 and S2) that characterizes the uncoupling of eNOS. eNOS uncoupling can lead to an increase in the levels of reactive nitrogen species and ROS [33]. After 24 h of incubation with 10 µM of Aβ isoforms, the eNOS monomer/dimer ratio did not change (Figure S1). At the 48 h time point, the eNOS monomer/dimer ratio increased in Aβ_42_-treated cells (Figure S2).

Thus, Aβ_42_ and iso-Aβ_42_ isoforms have the most pronounced effect on the distinctive redox parameters of bEnd.3 cells. The Aβ_42_ effect is most pronounced for the production of NO, and iso-Aβ_42_ induces the highest increase in the ROS.

### 2.3. Aβ Isoforms Affect Cytosolic and Mitochondrial Calcium in a Different Mode

**Figure 3:**
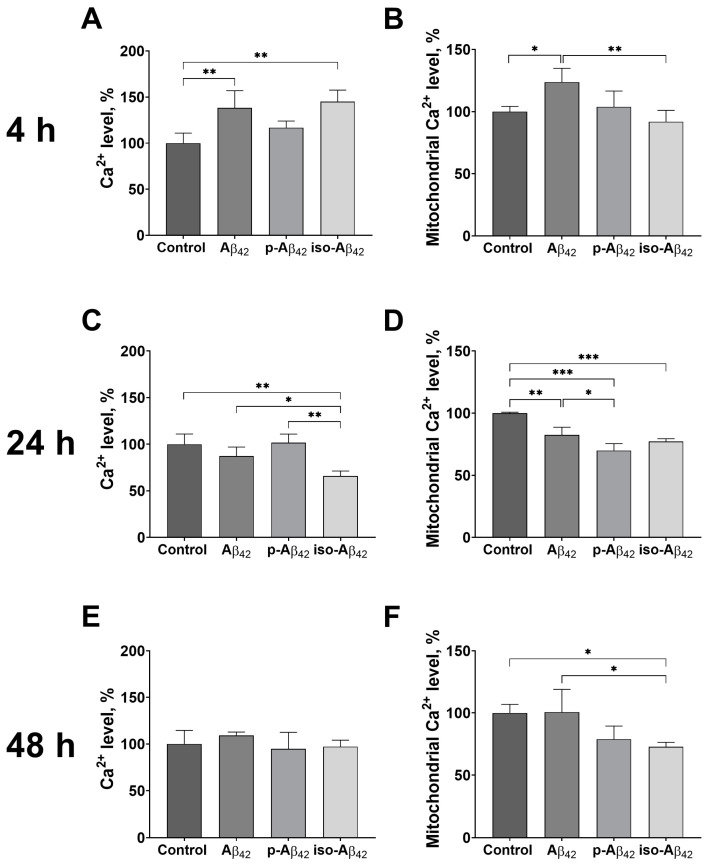
The effects of beta-amyloid isoforms on intracellular and mitochondrial calcium levels in bEnd.3 cells. Cells were incubated for 4 h (**A**,**B**), 24 h (**C**,**D**) and 48 h (**E**,**F**) with 10 μM of Aβ_42_, p-Aβ_42_ and iso-Aβ_42_. Flow cytometry was used to analyze changes in intracellular calcium (**A**,**C**,**E**) and mitochondrial calcium (**B**,**D**,**F**). All parameters were normalized to control. The values in the control samples were taken as 100%. The mean ± SD in 3 independent experiments in triplicates is shown in the figure. The statistical difference between experimental groups was analyzed by one-way analysis of variance using the post hoc Tukey test for multiple comparisons. *—*p* < 0.05, ** —*p* < 0.01 and ***—*p* < 0.001.

The observed NO and ROS increase induced by Aβ isoforms can be caused by an intracellular Ca^2+^ up-regulation due to the activation of endothelial nitric oxide synthase (NOS) [24] and NADPH oxidase [34], respectively. After 4 h of incubation, there was a cytosolic Ca^2+^ increase in Aβ_42_- and iso-Aβ_42_-treated bEnd.3 cells, while the level of mitochondrial Ca^2+^ increased (by 23%) only in the Aβ_42_-treated cells (Figure 3A,B).

After 24 h, the picture changed: in Aβ_42_-treated cells, the level of cytosolic Ca^2+^ reduced to the control level, while in iso-Aβ_42_-treated cells, the calcium level dropped by 35% below the control level (Figure 3C). The level of mitochondrial Ca^2+^ after 24 h of incubation was down-regulated throughout the conditions: a 15% decrease was observed in Aβ_42_-treated cells, and a 30% decrease was induced by a p-Aβ_42_ and iso-Aβ_42_ treatment (Figure 3D). After 48 h of incubation, the level of cytosolic Ca^2+^ in the samples treated with Aβ isoforms did not differ from the control (Figure 3E). At the same time, the level of mitochondrial Ca^2+^ in Aβ_42_-treated cells did not differ from the control and remained reduced in p-Aβ_42_- and iso-Aβ_42_-treated cells (Figure 3F).

### 2.4. Aβ_42_ and Iso-Aβ_42_ Induce an Increase in the Mitochondrial Potential in the Blood-Brain Barrier Cells

An increase in the cytosolic Ca^2+^ level can lead to a mitochondrial potential decrease due to a number of mechanisms, including the activation of proteins that form mitochondrial channels, in particular Bax and Bid due to the activation of calpains, as well as Bad and Bik due to the activation of calcineurin. In addition, mitochondrial megachannels may be activated [35]. The activation of calcium-sensitive phospholipase A2 can damage the mitochondrial membrane [36].

**Figure 4:**
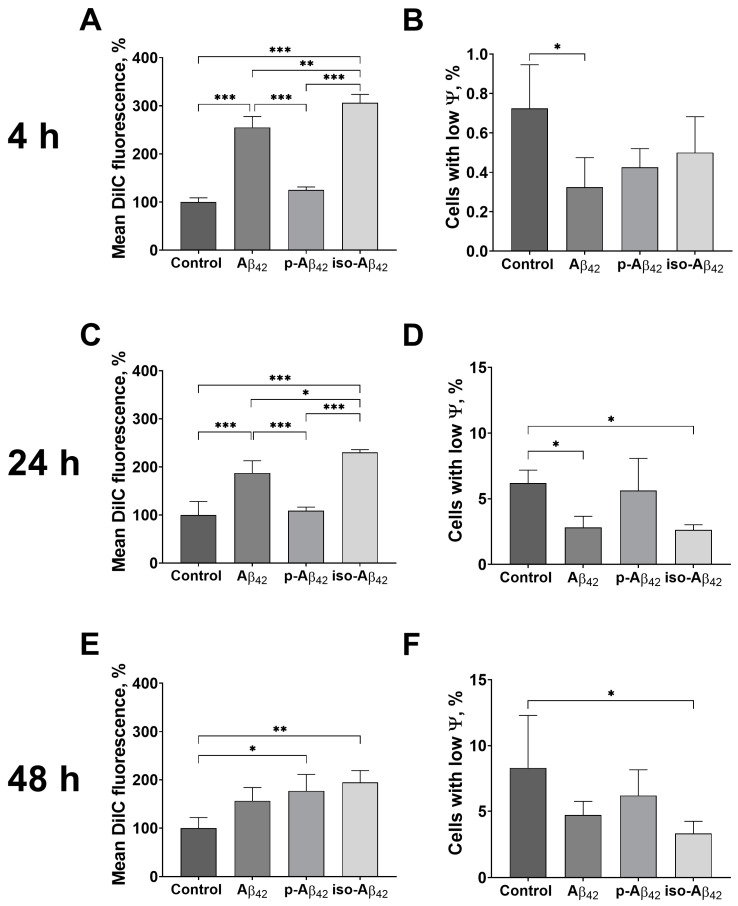
The effects of beta-amyloid isoforms on mitochondrial potential in bEnd.3 cells. Cells were incubated for 4 h (**A**,**B**), 24 h (**C**,**D**) and 48 h (**E**,**F**) with 10 μM of Aβ_42_, p-Aβ_42_ and iso-Aβ_42_. Flow cytometry was used to analyze changes in mean MitoProbe DilC fluorescence (**A**,**C**,**E**) and the number of cells with low mitochondrial potential (ψ) (**B**,**D**,**F**). Mean DilC fluorescence was normalized to control. The values in the control samples were taken as 100%. The mean ± SD in 3 independent experiments in triplicates is shown in the figure. The statistical difference between experimental groups was analyzed by one-way analysis of variance using the post hoc Tukey test for multiple comparisons. *—*p* < 0.05, **—*p* < 0.01 and ***—*p* < 0.001.

A four-hour treatment with Aβ_42_ and iso-Aβ_42_ of the BBB cells induced a 2.5- and 3-fold mitochondrial potential increase, respectively (Figure 4A). The p-Aβ_42_ treatment did not change the cell mitochondrial potential. An increase in the mitochondrial potential of the Aβ_42_-treated cells resulted in a decrease in the number of cells with a reduced mitochondrial potential (Figure 4B). After 24 h of incubation with Aβ_42_ and iso-Aβ_42_, the mitochondrial potential of the cells remained 1.9 and 2.3 times higher than the control (Figure 4C). Accordingly, the percentage of cells with a low mitochondrial potential in these cell populations was below the control. With an increase in the incubation time of up to 48 h, an increase in the mitochondrial potential of p-Aβ_42_-treated cells occurs and the iso-Aβ_42_ effect on the cells persists (Figure 4E).

### 2.5. Iso-Aβ_42_ Has the Most Rapid and Pronounced Effect on Mitochondrial Functioning in the Blood–Brain Barrier Cells

**Figure 5:**
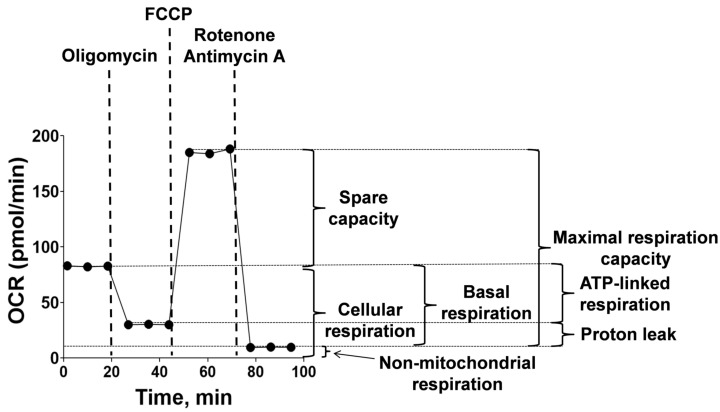
Scheme for measuring the bioenergetics parameters. Seahorse technology was used to assess cellular OCR. The ATP synthase inhibitor oligomycin, protonophore FCCP (carbonyl cyanide 4-(trifluoromethoxy)phenylhydrazone), and mitochondrial complex I and III inhibitors, Rotenone and antimycin A, respectively, were used to further determine indicators of cellular respiration. OCR is the oxygen consumption rate. ECAR is the extracellular acidification rate. The respiration parameters were calculated as follows: Basal respiration = cellular respiration—non-mitochondrial respiration; ATP-linked respiration = cellular respiration—oligomycin-inhibited respiration; Maximal respiration capacity = FCCP-induced respiration—non-mitochondrial respiration; Spare capacity = FCCP-induced respiration—cellular respiration; Proton leak = ATP-linked respiration—non-mitochondrial respiration.

**Figure 6:**
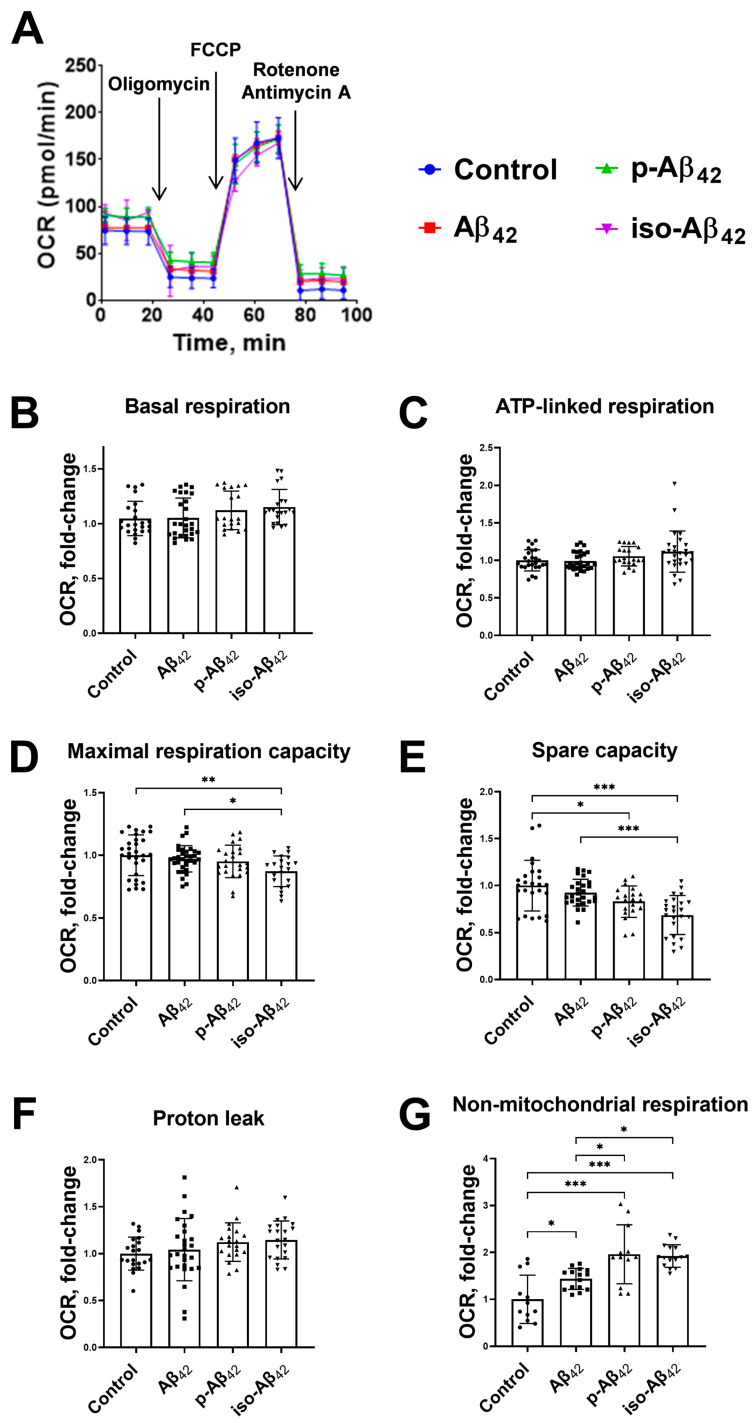
The effects of beta-amyloid isoforms on the bioenergetic functions of bEnd.3 cells after 4 h incubation. Cells were incubated with 10 µM of Aβ_42_, p-Aβ_42_ and iso-Aβ_42_. (**A**) Representative raw data of bioenergetic function. (**B**) Basal respiration, (**C**) ATP-linked respiration, (**D**) maximal respiration capacity, (**E**) spare capacity, (**F**) proton leak and (**G**) non-mitochondrial respiration are demonstrated. The results were calculated based on data obtained using bioenergetic functional analysis (for details, see Figure 5). The values in the control samples were taken as 1. All parameters were normalized to control. OCR is the oxygen consumption rate. FCCP is carbonyl cyanide 4-(trifluoromethoxy)phenylhydrazone. Each geometric figure (circle/square/up-pointing triangle/down-pointing triangle) in the histogram represents the result in an independent sample and corresponds to the Control, Aβ_42_, p-Aβ_42_ and iso-Aβ_42_. The mean ± SD in 3 independent experiments in 4–8 replications is shown in the figure. The statistical difference between experimental groups was analyzed by one-way analysis of variance using the post hoc Tukey test for multiple comparisons. *—*p* < 0.05, **—*p* < 0.01 and ***—*p* < 0.001.

**Figure 7:**
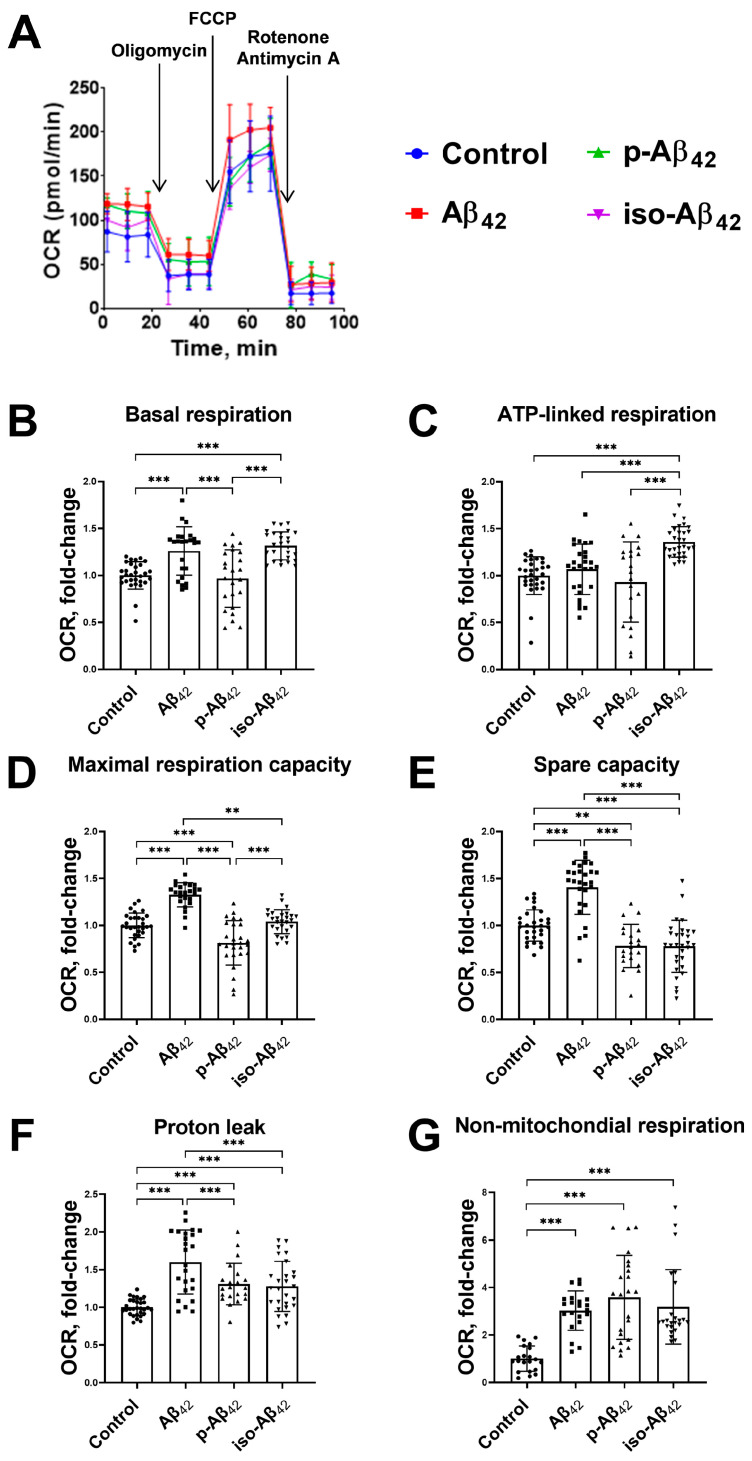
The effects of beta-amyloid isoforms on the bioenergetic functions in bEnd.3 cells after 24 h incubation. Cells were incubated with 10 µM of Aβ_42_, p-Aβ_42_ and iso-Aβ_42_. (**A**) Representative raw data of bioenergetic function. (**B**) Basal respiration, (**C**) ATP-linked respiration, (**D**) maximal respiration capacity, (**E**) spare capacity, (**F**) proton leak and (**G**) non-mitochondrial respiration are demonstrated. The results were calculated based on the data obtained from bioenergetic functional analysis (for details, see Figure 5). The values in the control samples were taken as 1. All parameters given are normalized to control. OCR is the oxygen consumption rate. FCCP is carbonyl cyanide 4-(trifluoromethoxy)phenylhydrazone. Each geometric figure (circle/square/up-pointing triangle/down-pointing triangle) in the histogram represents the result in an independent sample and corresponds to the Control, Aβ_42_, p-Aβ_42_ and iso-Aβ_42_. The mean ± SD in 3 independent experiments in 5–8 replications is shown in the figure. The statistical difference between experimental groups was analyzed by one-way analysis of variance using the post hoc Tukey test for multiple comparisons. **—*p* < 0.01 and ***—*p* < 0.001.

**Figure 8:**
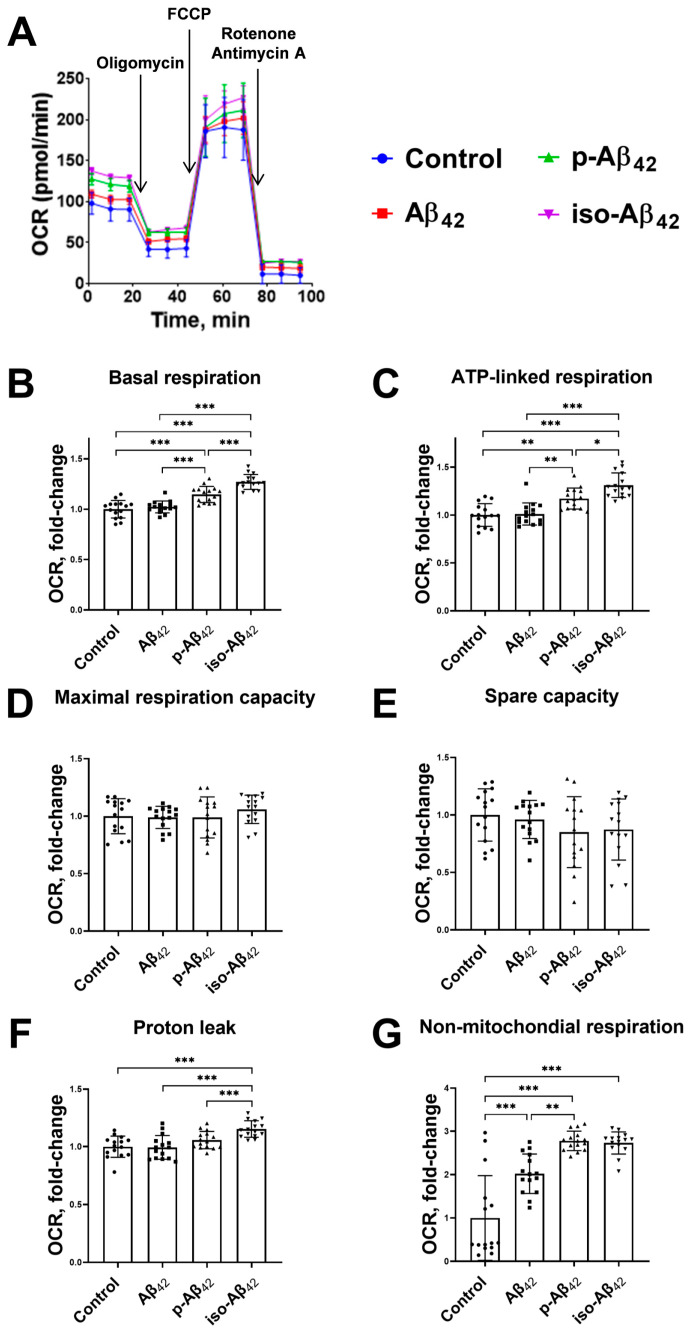
The effects of beta-amyloid isoforms on the bioenergetic functions in bEnd.3 cells after 48 h incubation. Cells were incubated with 10 µM of Aβ_42_, p-Aβ_42_ and iso-Aβ_42_. (**A**) Representative raw data of bioenergetic function. (**B**) Basal respiration, (**C**) ATP-linked respiration, (**D**) maximal respiration capacity, (**E**) spare capacity, (**F**) proton leak and (**G**) non-mitochondrial respiration are demonstrated. The results were calculated based on the data obtained from bioenergetic functional analysis (for details, see Figure 5). The values in the control samples were taken as 1. All parameters given are normalized to control. OCR is the oxygen consumption rate. FCCP is carbonyl cyanide 4-(trifluoromethoxy)phenylhydrazone. Each geometric figure (circle/square/up-pointing triangle/down-pointing triangle) in the histogram represents the result in an independent sample and corresponds to the Control, Aβ_42_, p-Aβ_42_ and iso-Aβ_42_. The mean ± SD in 3 independent experiments in 4–5 replications is shown in the figure. The statistical difference between experimental groups was analyzed by one-way analysis of variance using the post hoc Tukey test for multiple comparisons. *—*p* < 0.05, **—*p* < 0.01 and ***—*p* < 0.001.

**Figure 9:**
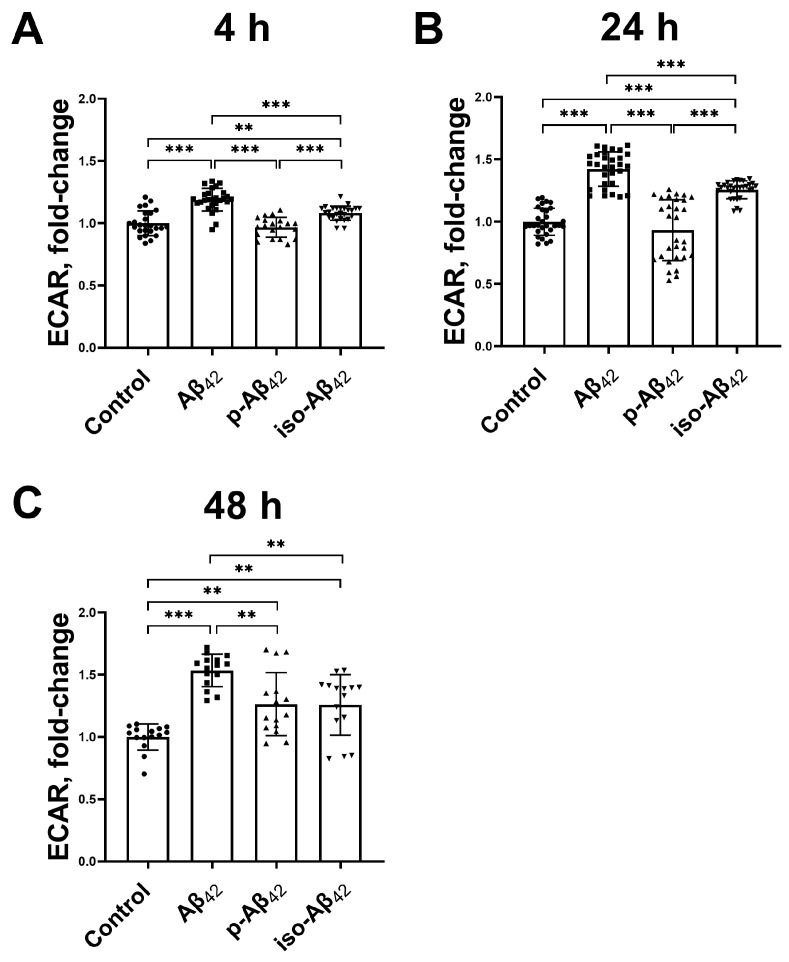
Effect of beta-amyloid isoforms on the extracellular acidification rate. Mouse brain endothelial cells bEnd.3 were incubated for (**A**) 4 h, (**B**) 24 h and (**C**) 48 h with 10 µM of Aβ_42_, p-Aβ_42_ and iso-Aβ_42_. Extracellular acidification rate (ECAR)—the level of acidification of the extracellular medium. The values in the control samples were taken as 1. The histograms indicate the ratio of values in samples with beta-amyloid isoforms, normalized to the control. Each geometric figure (circle/square/up-pointing triangle/down-pointing triangle) in the histogram represents the result in an independent sample and corresponds to the Control, Aβ_42_, p-Aβ_42_ and iso-Aβ_42_. The mean ± SD in 3 independent experiments in 5–8 replications is shown in the figure. The statistical difference between experimental groups was analyzed by one-way analysis of variance using the post hoc Tukey test for multiple comparisons. **—*p* < 0.01 and ***—*p* < 0.001.

The Aβ_42_ isoforms affect the mitochondrial potential in different modes, and the mitochondrial potential is directly related to the mitochondrial respiration and oxidative phosphorylation processes. A schematic representation of the assessment of the respiration indicators (Figure 5) and representative raw data of the bioenergetic function in bEnd.3 cells (Figure 6A, Figure 7A and Figure 8A) are given. The parameters of bEnd.3 cellular respiration, such as the level of basal respiration, ATP-linked respiration, maximal respiration capacity, spare capacity, proton leak, and non-mitochondrial respiration (Figure 5), were evaluated after 4, 24 and 48 h of incubation with 10 µM of Aβ_42_, p-Aβ_42_ and iso-Aβ_42_ (Figure 6, Figure 7 and Figure 8). The extracellular acidification rate (ECAR) was also assessed (Figure 9).

Basal respiration is the limit below which the cell is not able to maintain oxidative phosphorylation at the level covering the energy needs. A four-hour incubation with amyloid peptides did not result in an increase in the level of basal respiration (Figure 6B). Twenty-four-hour incubation caused a ~30% increase in the basal respiration with the addition of Aβ_42_ and iso-Aβ_42_, but not p-Aβ_42_ (Figure 7B). An increase in the incubation time up to 48 h resulted in a 17% up-regulation of the basal respiration which was induced by p-Aβ_42_, while a maximum increase in the basal respiration (27%) was observed in iso-Aβ_42_-treated cells (Figure 8B). At the same time, an Aβ_42_-related basal respiration returned to the control level (Figure 8B).

ATP-linked respiration defines how efficiently protons generated in the electron transport chain accumulate in the inner mitochondrial membrane and get used in ATP synthesis. A four-hour treatment with Aβ peptides did not induce the ATP-linked oxygen consumption rate (OCR) (Figure 6C), but 24 h of incubation with iso-Aβ_42_ promoted a ~30% increase (Figure 7C). After 48 h of incubation, an increase in the ATP-linked respiration (by 17%) also appeared in the p-Aβ_42_-treated cells, while in the presence of iso-Aβ_42_, the increase in this parameter was more pronounced (31%) (Figure 8C). The increase in ATP-linked respiration may be associated with the cell requirements in additional ATP.

The maximal respiration capacity decreased by ~13% after 4 h of incubation with iso-Aβ_42_ (Figure 6D) and was restored after 24 h (Figure 7D). Aβ_42_ and p-Aβ_42_ did not affect the maximal respiration capacity after 4 h of incubation (Figure 7D), but 24 h of incubation with these peptides induced changes in different modes: Aβ_42_ up-regulated this parameter by ~30%, and p-Aβ_42_ reduced it by ~19% (Figure 7D). A low maximal respiration capacity may indicate a decrease in the availability of the substrate or a violation of the integrity of mitochondria [37]. With an increase in the incubation time to 48 h, the values of the maximal respiration capacity induced by Aβ_42_ isoforms returned to the control values (Figure 8D).

A spare capacity reflects the difference between the maximal respiration capacity and basal respiration. We found that the spare capacity decreased during 4 h of incubation with p-Aβ_42_ and iso-Aβ_42_, and this effect persisted after 24 h (Figure 6E and Figure 7E). In the presence of Aβ_42_, this parameter of cellular respiration increased by ~40% after 24 h (Figure 7E). One of the reasons for the depletion in the spare capacity may be oxidative stress [38]. After 48 h of incubation, the spare capacity values in the samples treated with Aβ_42_ isoforms returned to the control values (Figure 8E).

An increase in the proton leak may be associated with a number of factors, including the increased activity of the uncoupling protein, and damage to the inner mitochondrial membrane and/or electron transport chain complexes. The result is a proton leak into the matrix and oxygen consumption in the absence of normal proton translocation across the inner mitochondrial membrane by complexes I, III and IV. None of the studied isoforms changed this parameter after 4 h (Figure 6F). Twenty-four-hour incubation resulted in an increase in the proton leak by 60%, 31% and 28% with the addition of 10 µM Aβ_42_, p-Aβ_42_ and iso-Aβ_42_, respectively (Figure 7F). With an increase in the incubation time to 48 h, a small but significant increase in the proton leak remained only in the samples treated with iso-Aβ_42_ (Figure 8F). It should be noted that the ATP-linked respiration and proton leak increased under oxidative stress [39,40].

Non-mitochondrial respiration in bEnd.3 cells treated with all Aβ isoforms was elevated. After 4 h of incubation, Aβ_42_, p-Aβ_42_ and iso-Aβ_42_ up-regulated the non-mitochondrial respiration by 43%, 96% and 92%, respectively (Figure 6G). An increase in the incubation time to 24 h led to an even greater effect of the Aβ isoforms: Aβ_42_, p-Aβ_42_ and iso-Aβ_42_ induced growth in the non-mitochondrial respiration till 300%, 358% and 260%, respectively (Figure 7G). After 48 h of incubation, an increase in the non-mitochondrial respiration was 200%, 277% and 273% for Aβ_42_, p-Aβ_42_ and iso-Aβ_42_, respectively, compared to the control (Figure 8G).

The extracellular acidification rate may be associated with increased glycolysis or non-glycolytic acidification. After 4 h of incubation with Aβ_42_ and iso-Aβ_42_, but not p-Aβ_42_, the extracellular acidification rate elevates (Figure 9A,B), which indicates a possible partial increase in glycolysis in cells. This effect develops over time, and after 24 h in the presence of Aβ_42_ and iso-Aβ_42_, a more significant acidification of the medium is observed, while p-Aβ_42_ still does not affect this parameter. With an increase in the incubation time to 48 h, the up-regulation of the ECAR is observed in the presence of all isoforms, and the maximum increase (53%) is in the Aβ_42_-treated cells (Figure 9C).

Thus, under the action of Aβ peptides, first of all, spare capacity decreases and glycolysis is activated, and then the basal respiration is activated, and the proton leak increases. The most rapid and pronounced changes in the functioning of the mitochondria were induced under the action of iso-Aβ_42_, the least pronounced under the action of p-Aβ_42_. At the same time, the nature of the change in the basal respiration and acidification (ECAR) of the medium after 24 h is similar to the change in the mitochondrial potential.

## 3. Discussion

Changes, associated with the dysfunction of the BBB, in the balance between the transport of Aβ from the blood to the brain and back [5,11,12] can lead to the accumulation of Aβ in the brain and the progression of AD. The question of the effect of the various blood-borne Aβ forms on the BBB remains open. The main BBB functional element is represented by endothelial cells, creating a barrier and providing an Aβ exchange between the blood plasma and the brain. The BBBs’ dysfunction in AD is accompanied by both functional changes, such as an increase in its permeability, and alterations in the intracellular parameters of the BBBs cells.

We evaluated the effect of Aβ isoforms on the redox status of endothelial cells and the mitochondrial functioning. bEnd.3 cells are polarized, and, in our model, we added Aβ isoforms to the luminal side to simulate the action of blood-borne Aβ species. Post-translational Aβ modifications, such as isomerization and phosphorylation, significantly alter the effect of Aβ on neuronal cells [24,25,26,27,32,41] and are an important factor in AD, but their effect on endothelial cells has not been previously studied. Maintaining the redox status of the BBBs cells is essential for the normal functioning of the BBB [42,43,44]. The cell redox status is closely related to the mitochondrial health, strongly affecting the cell functioning both under normal and pathological conditions in endothelial cells [45].

In accordance with the previously reported data for 9 μM of Aβ_42_ [15], 10 μM of Aβ_42_ do not have a cytotoxic effect on bEnd.3 cells after 48 h of incubation (Figure 1). The similar absence of toxicity was observed for p-Aβ_42_. On the contrary, iso-Aβ_42_ causes an increase in the percentage of dead cells after 48 h of incubation, which correlates with its increased toxicity to neuronal cells [15,24].

It is important to note that in contrast to neuronal cells, conventional Aβ targets, BBB endothelial cells implement the transport of Aβ from the blood to the brain and vice versa by transcytosis [46,47], and the amount of Aβ marked for degradation in these cells is extremely low [48]. It may result in the differences in the effect of Aβ_42_ peptides on the BBBs cells compared to the brain cells which we detected. Despite the absence of a toxic effect, Aβ_42_ isoforms significantly change the redox status of the cells of the BBB, leading to an increase in the levels of NO, ROS and GSH after 4 h of incubation (Figure 2A,D,G). It is known that in neuronal cells, Aβ_42_ to Na,K-ATPase binding induces intracellular signaling and leads to a rapid change both in the ROS and in the glutathione level [16]. We assume that the reason for the redox status change in endothelial BBB cells is also the binding of amyloid to receptors. Once the incubation time increases to 48 h, these changes persist or get stronger (Figure 2C,F,I). The most significant development of oxidative stress, characterized by an increase in the ROS level, is observed in the case of iso-Aβ_42_ (Figure 2G–I) and, apparently, is the cause of the greatest toxicity of this isoform. Iso-Aβ_42_, associated with a higher toxicity and ROS level for neuronal and neuron-like cells [32], also demonstrated this effect in the BBBs cells (Figure 2G–I). The effect of p-Aβ_42_ on ROS, on the contrary, proved itself to be later than for Aβ_42_ and iso-Aβ_42_ (Figure 2G–I). Thus, the ROS’ activation is an ultimate response to the amyloid treatment both in the neurons and in endothelial BBB cells. The advanced formation of ROS in the BBBs cells is a sign of vascular abnormalities [49]. The growth of ROS can lead to an increase in the barrier’s permeability and degradation of tight junction proteins [44]. An increase in ROS and iso-Aβ_42_-induced cytotoxicity both conclude that iso-Aβ_42_ can disrupt the BBB more than the other Aβ isoforms. One of the distinctive Aβ_42_ effects on brain neuronal cells is a decrease in the level of reduced glutathione [24,50,51,52]. In the BBBs cells, those treated with Aβ_42_ isoforms, the GSH, on the contrary, increases. In bEnd.3 cells, the GSH level was more responsive to Aβ_42_ and iso-Aβ_42_ at shorter incubation times and replicated ROS patterning (Figure 2D–F). The glutathione defensive system acts as a compensatory mechanism and may be activated in response to the up-regulation of ROS. The increase in the GSH reflects the greater resistance of the BBBs cells to oxidative stress compared to neuronal cells. As a system prone to oxidative stress, the BBBs endothelial cells have elevated the GSH, glutathione peroxidase, glutathione reductase and catalase levels compared to other brain cells [53]. GSH is also important for the transport of various substances across the BBB [54]. The GSH and ROS increase creates favorable conditions for protein glutathionylation, which affects their functioning [55]. Thus, S-glutathionylation of the mitochondrial Complex I and Complex II is possible [56]. This, in turn, can affect the ROS level produced by these complexes [56,57].

Aβ_42_ isoforms cause a strong NO increase in the BBBs cells. At the same time, Aβ_42_ is an absolute leader among isoforms in the induction of the growth of NO (Figure 2A–C). According to our data, the growth of NO during 24 h of incubation is not connected with eNOS uncoupling (Figure S1). P-Aβ_42_ induces the growth of NO later than Aβ_42_ and iso-Aβ_42_, and it is less pronounced (Figure 2A–C). A simultaneous increase in ROS and NO can lead to the reorganization of the cytoskeleton of endothelial cells [35]. A NO and O_2_^●^-interaction leads to the formation of peroxynitrite, which may cooperate with O_2_ to uncouple eNOS and amplify the ROS production [45]. At low ROS concentrations, NO protects cells, but at high ROS concentrations, NO can enhance their damaging effect [58,59,60,61]. An increase in the uncoupling of eNOS in Aβ_42_-treated cells after 48 h of incubation (Figure S2) may be induced by a maximum NO growth in the cells treated with this isoform, which together with the ROS growth after 48 h of incubation, significantly affects the eNOS uncoupling. The production of NO is closely related to the state of mitochondria, which can consume NO or produce it themselves using mitochondrial NOS [58,59,60,61]. NO stimulates mitochondrial biogenesis and influences the mitochondrial respiration [62]. In addition, NO stimulates the production of ROS and reactive nitric species by mitochondria. On the other hand, being a potent vasodilator throughout the body, NO increases the O_2_ supply to the mitochondria [63].

Changes in the cell redox status may be caused by a calcium increase, detected in many cell types under an Aβ treatment [15,64,65,66,67,68]. The cytoplasmic Ca^2+^ concentration is one of the key factors in the regulation of the paracellular permeability, affecting the tight junction and cytoskeletal proteins [35,69]. In Aβ_42_- and iso-Aβ_42_-treated bEnd.3 cells, we observe a short-term Ca^2+^ increase (Figure 3A), overlapping on the timeline with the increase in the ROS. An elevation in the Ca^2+^ level leads to the activation of NADPH oxidase [35], which may be the reason for the ROS level increase. On the other hand, ROS activates the oxidant-sensitive transient receptor potential (TRP) channels in the endothelium [70]. However, the cytosolic Ca^2+^ level returns to the control with an increase in the exposure time, while the ROS level remains high. Thus, Ca^2+^ can cause an initial ROS increase, while other mechanisms are responsible for maintaining ROS at a high level. The activation of endothelial NO synthase, leading to an increase in NO, may be both Ca^2+^-calmodulin-dependent [34] and Ca^2+^-independent [71]. However, the maximum NO increase is observed for Aβ_42_, while the Ca^2+^ levels for Aβ_42_ and iso-Aβ_42_ are similar (Figure 2A–C and Figure 3A,C,E). In addition, a high NO level, in contrast to Ca^2+^, persists for a long time. It can be assumed that a prompt cytosolic Ca^2+^ wave is a trigger of the activation of NO but is dispensable for the maintenance of NO, and a long-term NO increase is not explained by the Ca^2+^ up-regulation.

The ROS’ increase and NOs growth are closely related to the state of mitochondria [39,63]. According to our data, the mitochondrial potential of Aβ isoforms-treated BBB cells increases (Figure 4), and the profile of the changes in the mitochondrial potential for Aβ isoforms is similar to the patterning of the changes in the cell redox parameters since, in this case, p-Aβ_42_ is also characterized by the least effect. In contrast to the BBBs cells, Aβ in neuronal cells leads to a decrease in the mitochondrial potential [15,24], associated with the induction of cell death.

Aβ_42_ isoforms significantly alter the cellular respiration represented by the sum of the basal mitochondrial and non-mitochondrial oxygen consumption. The maximal respiration capacity consists of the sum of the basal respiration and spare capacity. The basal respiration, in turn, is made up of an ATP-linked respiration and proton leak (Figure 5) [38,72]. According to our data, in bEnd.3 cells, if we accept the maximal respiration capacity as 100%, then the spare capacity is about 59%, the ATP-linked respiration is 33%, the proton leak is 9%, and the non-mitochondrial respiration is 9% (the basal respiration is 42%) (Figure 6A and Figure 8A). This correlates well with the data for bovine aortic endothelial cells [49] and differs significantly from the values of mitochondria-rich hepatocytes and cardiomyocytes, characterized by a higher spare capacity. In the cultures of primary neurons, compared with the BBBs cells, the spare capacity is lower (42%), the ATP-linked respiration is higher (46%), the proton leak is higher (12%), and the non-mitochondrial respiration is two times higher (19%) [49]. This indicates a greater need for ATP in neuronal cells compared to the BBBs cells. The difference in bioenergetics is explained by the different functional activity of the cells [49].

The twenty-four-hour exposure of bEnd.3 cells to Aβ_42_ and iso-Aβ_42_ results in an increase in the basal respiration, while p-Aβ_42_ does not affect it (Figure 7B). After 48 h of incubation, the Aβ_42_ effect on the basal respiration disappears, the p-Aβ_42_ effect appears, and the iso-Aβ_42_ effect persists (Figure 8B). The profiles of the amyloid isoforms-induced changes in the basal respiration and the mitochondrial potential are similar, however, a change in the mitochondrial potential precedes a respiration shift. Non-mitochondrial respiration occurs due to the interaction of oxygen with non-mitochondrial oxidases and is usually accompanied by an increase in cytosolic ROS [49]. Indeed, we observe a synchronous increase in the non-mitochondrial respiration (Figure 6G and Figure 8G) and the ROS level (Figure 2G,I), all induced by Aβ isoforms.

An increase in the proton leak after 24 h of incubation with Aβ isoforms may be associated with (1) an increase in the activity of the uncoupling protein, (2) damage to the inner mitochondrial membrane, (3) damage to electron transport chain complexes (4) and electron slippage [38,49]. Since the mitochondrial potential does not decrease but rather increases, the damage to the inner mitochondrial membrane is excluded in our case. It is possible that an increase in the proton leak is induced by the simultaneous growth of NO and ROS. For example, the synchronous exposure of BAEC cells to ROS and NO donors results in a proton leak [39].

ATP-linked respiration does not change after an exposure to Aβ_42_ and p-Aβ_42_, but increases after 24 h of incubation with iso-Aβ_42_ (Figure 7C). After 48 h of incubation, the ATP-linked respiration increases in the cells treated with both p-Aβ_42_ and iso-Aβ_42_ (Figure 8C). The maximum effect is achieved in iso-Aβ_42_-treated cells (Figure 8C). This is explained by the need for additional ATP synthesis due to the iso-Aβ_42_ cytotoxic effect. The maximal respiration capacity induced by iso-Aβ_42_ decreases after 4 h of incubation (Figure 6D), and this is a consequence of a spare capacity decrease (Figure 6E and Figure 7E), which remains low after 24 h (Figure 7C). At the same time, the maximal respiration capacity no longer differs from the control due to the increase in the basal respiration. For p-Aβ_42_, the spare capacity is reduced after a 4- and 24-hour incubation (Figure 6E and Figure 8E), which leads to a decrease in the maximal respiration capacity after 24 h. For Aβ_42_, on the contrary, the spare capacity and maximal respiration capacity increase (Figure 7D). The spare capacity is probably reduced due to oxidative stress [39], but the Aβ_42_-treated cells appear to be protected by a high NO level. The maximal respiration capacity depends on the substrate availability, mitochondrial mass, and electron transport chain integrity [49]. After 48 h of incubation, the spare capacity and maximal respiration capacity return to the control values (Figure 8C,D), and it may be evidence of the cell adaptation.

Aβ_42_ and iso-Aβ_42_ treatments lead to extracellular acidification after 4 h of incubation, which further develops after 24 and 48 h (Figure 9). This increase may be a consequence of both the activation of glycolytic processes and NO growth, its further oxidation and acid formation. Indeed, the largest ECAR increase is caused by an exposure to Aβ_42_, also characterized by the highest NO against other isoforms (Figure 2A–C). Iso-Aβ_42_ has a weaker effect (regarding NO and ECAR), whereas no change in these parameters is observed after 4 h of exposure to p-Aβ_42_. After 24 h, the effects of Aβ_42_ and iso-Aβ_42_ on NO and the ECAR persist. Regarding the effects of p-Aβ_42_, there is an increase in the NO level but not in ECAR. Thus, the increase in the ECAR is probably explained by the activation of glycolysis.

The activation of cell bioenergetics and cellular respiration may be caused by the need for a more efficient Aβ transport. Since the activation of eNOS stimulates transcytosis [73], one can conclude that a NO increase, which is most pronounced for Aβ, is also aimed at the enhancement of transcytosis. A decrease in the availability of endothelial NO leads to an increase in the concentration of Aβ in the brain vessels, along with compensatory mechanisms for the vascular system’s protection [74]. According to the molecular dynamics data, the native form of Aβ_42_ shows a stronger interaction with the RAGE compared to p-Aβ_42_ and iso-Aβ_42_ [75]. The binding of some ligands to the RAGE can regulate eNOS [76]. This may be the reason for the maximum NO increase induced by Aβ_42_.

The assessment of the effects of the isoforms of Aβ has shown that p-Aβ_42_ has the least effect on the redox parameters and respiratory activity of the cells, while iso-Aβ_42_, which causes maximum oxidative stress, is the most toxic isoform. Iso-Aβ_42_ also changes the mitochondrial potential the most and disrupts the mitochondrial respiration parameters faster than other isoforms. The distinctive molecular mechanism behind these differences may be explained by the interaction of the isoforms with receptors or by their effect on ion transporters. p-Aβ_42_ has a lower and iso-Aβ has a higher oligomerization rate in the presence of divalent metal ions compared to Aβ [26,77]. This property may play an important role in the interaction of these isoforms with receptors. For instance, p-Aβ_42_, in contrast to Aβ_42_, does not cause the inhibition of Na,K-ATPase [26]. Moreover, the isomerization of Aβ_42_ leads to the stronger inhibition of the α7 nicotinic acetylcholine receptor [25], and this receptor is expressed in the membrane of the brain’s endothelial cells [78]. The distinctive action of p-Aβ_42_ and iso-Aβ_42_ is in line with the different amyloidogenicity of these isoforms. Iso-Aβ_42_ enhanced the amyloidogenicity in a mouse model of AD [20], whereas a p-Aβ_42_ injection reduced the rate of the amyloid plaques’ formation [26].

Our findings suggest that in mouse BBB cells, the effects of beta-amyloid peptides on mitochondria are closely associated with changes in the cell redox status. We found that Aβ_42_ and iso-Aβ_42_ caused a significant increase in NO, ROS, GSH, cytosolic calcium, the mitochondrial potential and the activation of glycolysis after 4 h of incubation. P-Aβ_42_ does not have such an effect, or it occurs much later. All isoforms activate the respiratory activity of mitochondria, while the phosphorylated isoform affects the cellular respiration parameters less than unmodified and isomerized forms do. The isomerized form has the highest cytotoxicity, causing maximum oxidative stress. Therefore, our data suggest that post-translational modifications of Aβ_42_ alter the amyloid effect on the BBBs cells and iso-Aβ_42_ acts as a more pathogenic isoform.

## 4. Materials and Methods

### 4.1. Synthetic Peptides′ Preparation

To prepare the 3 Aβ isoforms, cold hexafluoroisopropanol (Fluka) was added to dry Aβ_42_ (LifeTein, USA) [H2N]-DAEFRHDSGYEVHHQKLVFFAEDVGSNKGAIIGLMVGGVVIA-[COOH], p-Aβ_42_ (Biopeptide, San Diego, CA, USA) and iso-Aβ_42_ (LifeTein, Hillsborough, NJ, USA) peptides to a concentration of 1 mM. The incubation lasted for 60 min at room temperature [79]. Then, the peptide solutions were incubated on ice for 10 min and aliquoted into non-siliconized microcentrifuge tubes (0.56 mg of peptide per tube). The peptides in the tubes were dried under a vacuum using Eppendorf Concentrator 5301 (Hamburg, Germany). The dried peptides were stored at −80 °C. A total of 2.5 mM of peptide stock solution was prepared by adding 20 μL of 100% anhydrous dimethyl sulfoxide (DMSO; Sigma-Aldrich, St. Louis, MO, USA) to 0.22 mg of peptide and incubating for 1 h at room temperature. The peptides were further diluted to the required concentration with a buffer solution. The equivalent amount of DMSO was added to the control samples in all experiments. Only freshly prepared peptide solutions were used for all experiments. As shown by us earlier [80] by dynamic light scattering and turbidity measurements, these Aβ_42_ solutions do not contain particles in the ranges of 0.6–10 nm and 1–100 nm. Monomers constituted 80% in the preparation of Aβ_42_ [80].

### 4.2. Cell Culture

Mouse brain endothelial cells bEnd.3 (CRL-2299) from the American Type Culture Collection were cultured in Dulbecco’s Modified Eagles Medium (DMEM; Gibco, ThermoFisher Scientific, Waltham, MA, USA) containing 10% fetal bovine serum (FBS; Gibco, USA), 100 units/mL of penicillin, 100 µg/mL of streptomycin (Sigma, St. Louis, MO, USA), sodium pyruvate and GlutaMax (Gibco, ThermoFisher Scientific, Waltham, MA, USA) in T-25 and T-75 culture flasks at 37 °C in a humid atmosphere with 5% CO_2_ (the passages did not exceed 20).

### 4.3. Flow Cytometry

bEnd.3 cells were seeded on 24-well plates (Eppendorf, Germany) and cultured in a DMEM to a 90–95% confluence. Before the experiment, the cells were washed with serum-free Opti-MEM. The Aβ peptides from a stock solution (2.5 mM) in DMSO were dissolved in serum-free Opti-MEM (Gibco, ThermoFisher Scientific, Waltham, MA, USA) to a final concentration of 10 μM. An equivalent amount of DMSO was added to the control samples. The cells were incubated with Aβ isoforms at a concentration of 10 µM for 4 h, 24 h and 48 h. Twenty-four hours after the start of the incubation, FBS was added to a final concentration of 2.5%.

The levels of ROS, NO and GSH were assessed using fluorescent dyes dihydrorhodamine 123 (DHR123; ThermoFisher Scientific, Waltham, MA, USA), DAF-FM DA (ThermoFisher Scientific, Waltham, MA, USA) and ThiolTracker Violet (ThermoFisher Scientific, Waltham, MA, USA), respectively. To assess the level of ROS, DHR123 (Ex/Em = 507/525 nm) was added to the cells to a final concentration of 10 µM, as described by us previously [81,82]. The NO level was assessed by staining the cells with 5 μM of DAF-FM DA (Ex/Em = 495/515 nm), as described by us previously [83]. To assess the level of reduced glutathione, the cells were stained with 15 μM of ThiolTracker Violet (Ex/Em = 405/525 nm) [24]. MitoProbe DilC dye (Ex/Em = 638/658 nm; ThermoFisher Scientific, Waltham, MA, USA) was used at a ratio of 1:200 [81] for the mitochondrial potential evaluation. The mean fluorescence of the MitoProbe DilC and the percentage of cells with a reduced mitochondrial potential were assessed. The level of Ca^2+^ in the intact cells was assessed using the dye fluo-4 (Ex/Em = 494/516 nm; ThermoFisher Scientific, Waltham, MA, USA), adding it to the cells at a concentration of 2.5 μM according to [24,81]. A cell staining was performed in a 24-well plate for 30 min in the dark in a CO_2_ incubator (37 °C, 5% CO_2_). The mitochondrial Ca^2+^ level was assessed by cell staining with 5 μM of Rhod-2 AM dye (Ex/Em = 552/581 nm; ThermoFisher Scientific, Waltham, MA, USA).

After an incubation with the dyes, the cells were washed with Versene solution and detached by incubation with TryplE Express (Gibco, ThermoFisher Scientific, Waltham, MA, USA) in a CO_2_ incubator. Then, Opti-MEM with FBS at a concentration of 2.5% was added.

The percentage of dead cells in the population was estimated using propidium iodide (Ex/Em = 535/617 nm; Sigma, St. Louis, MO, USA) [84]. Propidium iodide was added to the cells at a concentration of 10 μg/mL for one minute prior to the analysis on a flow cytometer. In the analysis of the intracellular parameters, only cells with a whole membrane were taken into account, which are not stained with propidium iodide. The cell samples were analyzed on a BD LSR Fortessa flow cytometer (Becton Dickinson, Franklin Lakes, NJ, USA).

### 4.4. Estimation of the Cellular Bioenergetic Parameters

The parameters of the mitochondrial respiration, non-mitochondrial respiration and the ECAR were assessed using a Seahorse XFe24 Analyzer (Agilent, Santa Clara, CA, USA). To measure the cellular respiration parameters, bEnd.3 cells were seeded into the wells of an XF24 V7 plate (Agilent, Santa Clara, CA, USA) 4 days before the amyloid treatment, 8 thousand per well. The cells were counted using a CASY cell counter (Cambridge Bioscience, Cambridge, UK) according to the manufacturer’s protocol. By the beginning of the experiment, the cells reached confluency.

The respiration parameters were assessed using a cell respiration analysis kit (Seahorse XF Cell Mito Stress Test Kit, Agilent, Santa Clara, CA, USA), based on the published data in bEnd.3 cells [15,72]. The cells were incubated with 10 μM of Aβ_42_, p-Aβ_42_, iso-Aβ_42_ or 0.8% DMSO (control) in Opti-MEM without FBS for 4, 24 or 48 h in a CO_2_ incubator (37 °C, 5% CO_2_). The cells were washed with an assay media (pH 7.4; Agilent, Santa Clara, CA, USA), containing 25 mM of glucose, 1× GlutaMax and 2 mM of sodium pyruvate (Gibco, ThermoFisher Scientific, Waltham, MA, USA). Then, 500 µL of the assay media were added per well and incubated for 1 h at 37 °C without CO_2_.

The cellular oxygen consumption was determined, as well as the oxygen consumption after the addition of inhibitors (Figure 5). The final concentrations of the inhibitor solutions diluted in the assay media were: 1 μM of oligomycin (an inhibitor of ATP synthase), 0.5 μM of FCCP (a protonophore), 1 μM of rotenone and 1 μM of antimycin A (the inhibitors of complex I and III of mitochondria, respectively) [85,86]. A total of 50 μL of the inhibitor solutions were added to the empty wells provided for this purpose. The ECAR was also assessed. The results were analyzed using the Wave software (Agilent, Santa Clara, CA, USA) and GraphPad Prism 9.1.2 software (GraphPad Software Inc., San Diego, CA, USA). The basal respiration, maximal respiration capacity, ATP-linked respiration, spare capacity, proton leak and non-mitochondrial respiration were calculated as in [72].

### 4.5. Western Blot

The cells were incubated with 10 μM of Aβ_42_, p-Aβ_42_, iso-Aβ_42_ or 0.8% DMSO (control) in Opti-MEM without FBS for 24 or 48 h in a CO_2_ incubator (37 °C, 5% CO_2_). Then, the cells were lysed in the RIPA-buffer (25 mM tris-HCl, pH 7.6, 150 mM NaCl, 1% Nonidet-P40, 0.1% SDS and 1% sodium deoxycholate; ThermoScientific MA, USA, 34096) containing the protease inhibitors cocktail (Roche, Indianapolis, IN, USA, 11836145001) and the phosphatase inhibitors cocktail (Roche, Indianapolis, IN, USA, 4906837001) with stirring at 4 °C for 1 h. The lysates were then centrifuged at 16,100× g for 10 min at 4 °C and the supernatant was collected. The cell lysates were separated on 10% SDS PAGE electrophoresis and transferred to a PVDF-membrane (Bio-Rad, Hercules, CA, USA, 1620137). The membrane was blocked in 5% nonfat milk in TBST (50 mM of Tis-HCl, pH 7.4, 150 mM of NaCl and 0.1% Tween-20), and incubated with primary mouse antibodies to eNOS (anti-eNOS N30020L14, Becton Dickinson Transductional Laboratories, Franklin Lakes, NJ, USA) in TBST overnight at +4 °C. Then, the membrane was incubated with HRP-conjugated secondary antibodies (ThermoFisher Scientific, Waltham, MA, USA, A16078) and imaged with the chemiluminescence SuperSignal™ West Femto Maximum Sensitivity Substrate kit (ThermoFisher Scientific, Waltham, MA, USA, 34096) using a Bio-Rad ChemiDoc MP instrument (Bio-Rad, Hercules, CA, USA). The densitometric analysis was performed with the Image Lab 6.0.1 program (Bio-Rad, Hercules, CA, USA)

### 4.6. Statistical Data Analysis

All the experimental data are shown as the mean values ± standard deviations of the mean (SD), with the number of independent experiments indicated in the Figure legends. The statistical difference between the experimental groups was analyzed by a one-way analysis of variance (ANOVA) with Tukey correction for multiple comparisons. Probability values (*p*) less than 0.05 were considered significant. A statistical analysis was performed using GraphPad Prism 9.1.2 software (GraphPad Software Inc, San Diego, CA, USA).

## Data Availability

Not applicable.

## Supplementary Materials

The following supporting information can be downloaded at: https://www.mdpi.com/article/10.3390/ijms24010183/s1.

## Abbreviations

Aβ	beta-amyloid
Aβ_42_	beta-amyloid 1–42
AD	Alzheimer’s disease
BBB	blood–brain barrier
DHR123	dihydrorhodamine 123
DMEM	Dulbecco’s Modified Eagles Medium
DMSO	dimethyl sulfoxide
ECAR	extracellular acidification rate
eNOS	endothelial nitric oxide synthase
FBS	fetal bovine serum
FCCP	carbonyl cyanide 4-(trifluoromethoxy)phenylhydrazone
GSH	reduced glutathione
iso-Aβ_42_	beta-amyloid 1–42, containing isomerized Asp7 residue
NO	nitric oxide
NOS	nitric oxide synthase
OCR	oxygen consumption rate
p-Aβ_42_	beta-amyloid 1–42, containing phosphorylated Ser8 residue
PI	propidium iodide
RAGE	receptor for advanced glycation end products
ROS	reactive oxygen species
ZO-1	zonula occludens-1
