# Supplementary material for: Distinct Effects of Beta-Amyloid, Its Isomerized and Phosphorylated Forms on the Redox Status and Mitochondrial Functioning of the Blood–Brain Barrier Endothelium
Source: Int J Mol Sci. 2022 Dec 22;24(1):183. doi: 10.3390/ijms24010183 (PMC9820675; doi:10.3390/ijms24010183)
Supplement: Supplementary file 1 [file ijms-24-00183-s001.zip › ijms-2089349-supplementary.pdf]

Supplementary materials

Distinct effects of beta-amyloid, its isomerized and phosphorylated forms on the redox status and mitochondrial functioning of the blood-brain barrier endothelium

Aleksandra V. Petrovskaya[1,#], Artem M. Tverskoi[1,#], Evgeny P. Barykin[1], Kseniya B. Varshavskaya[1], Alexandra A. Dalina[1], Vladimir A. Mitkevich[1,*], Alexander A. Makarov[1] and Irina Yu. Petrushanko[1]

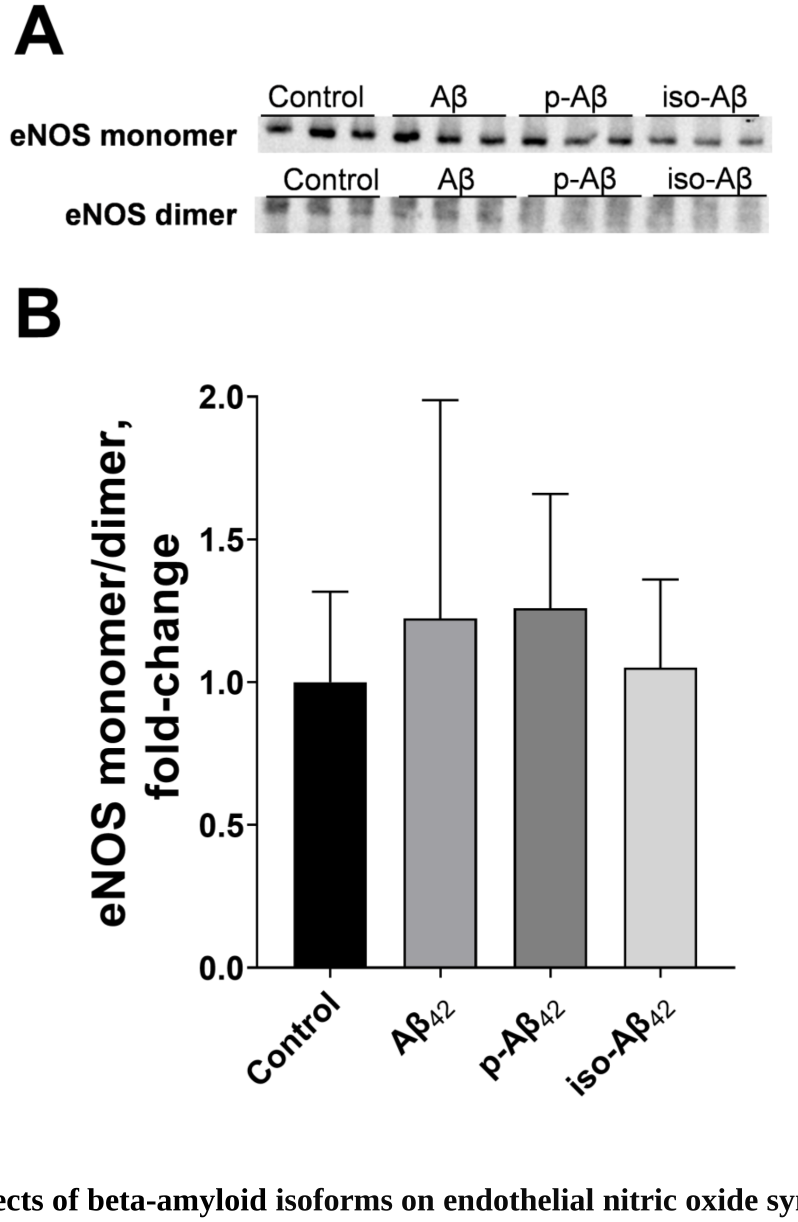

Figure S1. The effects of beta-amyloid isoforms on endothelial nitric oxide synthase (eNOS) monomer/dimer ratio in bEnd.3 cells. Cells were incubated for 24 hours with 10 μM of Aβ_{42}, p-Aβ_{42} and iso-Aβ_{42}. eNOS monomer/dimer ratio was measured with Western blot. **(A)** The representative blots and **(B)** the corresponding eNOS monomer/dimer ratio are presented. The

values in the control samples are taken as 1. Mean values ± SD from three independent experiments are shown.

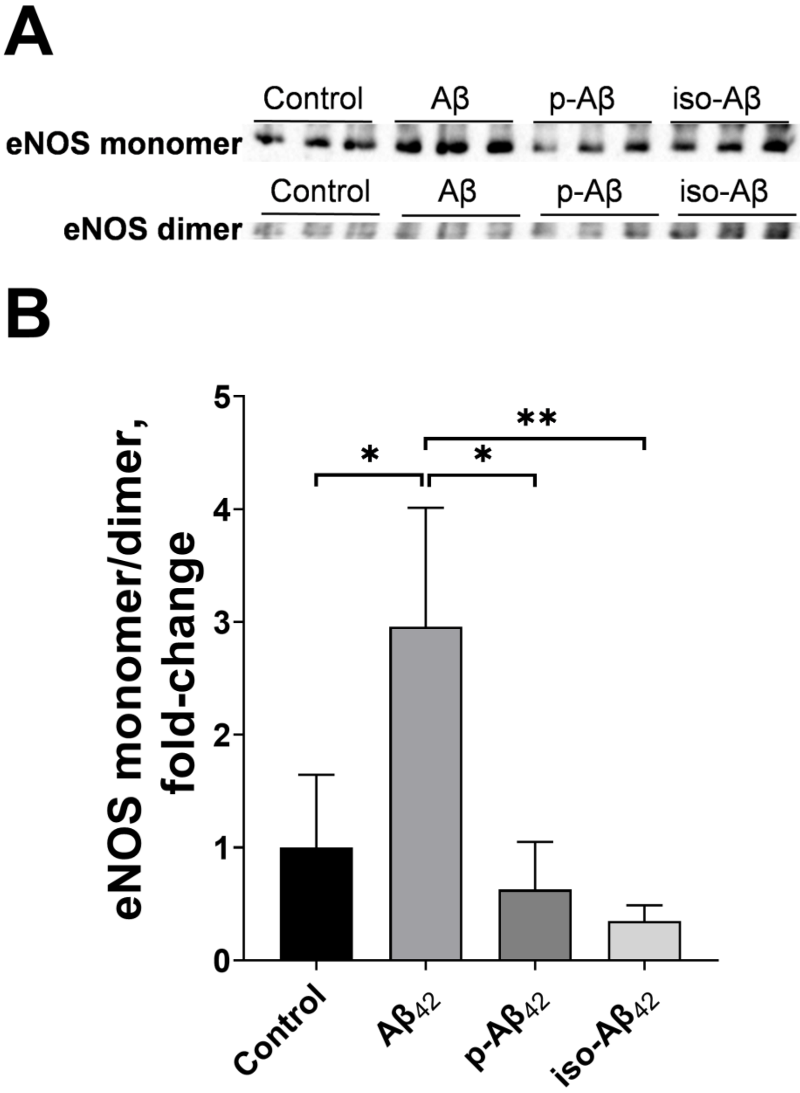

Figure S2. The effects of beta-amyloid isoforms on endothelial nitric oxide synthase (eNOS) monomer/dimer ratio in bEnd.3 cells. Cells were incubated for 48 hours with 10 μM of $A\beta_{42}$, p-$A\beta_{42}$ and iso-$A\beta_{42}$. eNOS monomer/dimer ratio was measured with Western blot. **(A)** The representative blots and **(B)** the corresponding eNOS monomer/dimer ratio are presented. The values in the control samples are taken as 1. Mean values ± SD from three independent experiments are shown. * - $p < 0.05$, ** - $p < 0.01$.
